# Research on E-Commerce Data Standard System in the Era of Digital Economy From the Perspective of Organizational Psychology

**DOI:** 10.3389/fpsyg.2022.900698

**Published:** 2022-05-04

**Authors:** Hongqiang Yue

**Affiliations:** Henan University School of Law/Intellectual Property School, Institute of Civil and Commercial Law of Henan University, Kaifeng, China

**Keywords:** virtual e-commerce, industrial agglomeration expansion, big data, e-commerce model, standard system

## Abstract

With the rapid development of technology and the economy, the expansion of the network has had a huge impact on the rapid expansion of the industrial agglomeration e-commerce industry, as well as ensuring the shopping experience of consumers. The rapid expansion of industrial cluster e-commerce has avoided precisely the limitations of logistical bottlenecks. Current networks and modern information technologies can provide good support and maintain a huge growth potential. In addition, digital technologies such as multimedia are becoming increasingly important in industry cluster marketing, and the concept of industry cluster e-commerce models is gaining more and more attention from companies. However, virtual e-commerce systems under industrial clusters have not been well researched in the existing studies. In this paper, through extensive research, literature reading and website browsing statistics, the virtual e-commerce models of different industrial agglomerations are studied. Firstly, the concept of big data and the processing of big data are given. Secondly, the concept of industrial agglomeration and the relationship between industrial agglomeration and e-commerce are analyzed. The basic number of domestic Internet users in the last 10 years is also counted, proving that the expansion of the Internet has led to a substantial growth of Internet users in the country and that e-commerce plays a significant role in the future of business activities. Finally the study concludes that different e-commerce models have different performance and roles in industrial agglomeration e-commerce and cannot be generalized. Instead, it is not good and can only develop different industrial agglomeration e-commerce models according to different environments.

## Introduction

In the long history of mankind, when people explore and discover the law of unknowns, they rely mainly on reasoning methods such as experience, theory, and assumptions, which are largely influenced by personal prejudice. Later, people invented mathematical tools such as statistics, sampling, and probability. Through careful design and extraction methods, a small number of data samples were obtained to infer the whole picture of things. Therefore, there are often deviations and distortions in understanding things. According to Victor Meyer, thanks to advances in technology, people can access all the data of a research object and understand things from different angles. Analyze the different dimensions of all data from an incomprehensible perspective. With the rapid expansion of electronic signal technology, e-commerce (Anam et al., 2017; Irene, 2018) has changed an inevitable outcome of the expansion of the times and is also a form of transaction that adapts to market demand. The expansion of e-commerce is very gratifying. After more than 10 years of expansion, B2C (Gui et al., 2019) and C2C (Navarro-Méndez et al., 2017) have become the main mode of e-commerce in China. The model has the vitality of information transparency, flexible trading, high efficiency and price advantage. With the rapid propagate of the Net, by the end of 2018, the number of Internet users in China reached 1.08 billion. A great deal of Internet users has established a good customer base for the expansion of e-commerce. In addition, the continuous improvement of relevant laws and regulations and the maturity of information technology have laid the foundation for the expansion of e-commerce. By combining big data with e-commerce, e-commerce based on big data will become the main research direction of the future society (Nik et al., 2017).

Mega data (big data) (Wang et al., 2017; Zhou et al., 2017) is what we often call big data, also known as massive data. Giant data is actually a data repository. In this era, it can be used as an asset. After professional analysis, the efficiency is higher, the amount of data is larger, the data is diverse, and the sources are different, most of which are instantaneous. The communication information generated during the sales process is also generated instantaneously. For example, customer basic data, website clicks, network data, etc., are all counted in big data, some are part of customer information, and some are not counted. In the 1980s, some scholars predicted big data and believed that big data will surely ignite the new wave of the third technological revolution. Since 2009, “Big Data” has made great progress with the rapid expansion of e-commerce and cloud computing (Liu et al., 2018) and is gradually becoming well known to the public. As can be seen from the latest data, the growth of data on the Internet and mobile Internet has gradually approached Moore’s Law, and global data and information have been created “over doubling every 18 months” over the years. The application of big data in industrial agglomeration (Xuan, 2017; Nádudvari et al., 2018) e-commerce is also getting more and more wide-ranging.

Industrial clusters (Cao et al., 2017; Wang and Yu, 2017) have a long history as well-functioning organizations. At the end of the 19th century, Marshall creatively defined the concept of “industry zone,” that is, industrial clusters. He defines “industrial zone” as the agglomeration of certain industrial zones, which is determined by two factors: history and natural resources. There are many companies of different sizes in the area. There is a close relationship between cooperation and competition, which gradually affects the integration of industrial clusters and society. According to Marshall, the reason for the emergence of “industry zones” in the region is a combination of inside and outside factors. Later, Weber believed that the phenomenon of industrial clusters was the result of regional and geographic influences. Companies with regional and geographic advantages have established close partnerships through partnerships with other related companies. Establish complex and close internal network relationships, achieve the aggregation of enterprises in a specific region, and then develop into industrial clusters. In recent decades, academia and industry have been highly involved in the expansion of synergies between industrial clusters and supply chains. They actively used industrial clusters and supply chains in corporate management (Heiner and Marc, 2018) and achieved remarkable results. Clusters and supply chains can provide a competitive advantage for businesses. However, with the rapid expansion of e-commerce, industrial clusters are faced with the dilemma of optimizing transformation and upgrading. The traditional approach to supply chain management is far from meeting the needs of users. Therefore, it is a major problem to study how e-commerce uses the first-mover advantage to promote synergy between industrial clusters and supply chains.

For the core enterprises in the industrial agglomeration, because of their own advantages in terms of capital and technology, as well as a number of strong manufacturers and suppliers, so that the online market established by the enterprise has a large number of members and good prospects for development, and attracts some new members to join, once the establishment of close cooperation in this online market, its members want to move to other online market will be very expensive, so that the core enterprises in the online market to consolidate their existing position (Yang et al., 2022; Han et al., 2021; Setiawan et al., 2022; Suska, 2022; Yu et al., 2022). Therefore, e-commerce has developed into a new opportunity to enhance the synergy of China’s supply chain and enhance its competitive advantage. In the end, this paper starts from the business reality of big data-based industry agglomeration e-commerce, fully considers the dependence of industrial agglomeration area on e-commerce in the era of big data, and studies the relationship between the concept of industrial agglomeration and the relationship between e-commerce and industrial agglomeration. Therefore, with the support of big data, this paper analyses the number of netizens, the level of economic expansion, etc., and compares the impact of e-commerce yield and industrial agglomeration e-commerce investment and big data and e-commerce on industrial agglomeration. The merits and demerits of e-commerce in the type of industrial agglomeration, and the expectation is to provide a summary and reference for the industry to gather e-commerce enterprises to obtain competitive advantages in the market competition.

## Big Data and E-Commerce Related Definitions

### Big Data Overview

With the popularity of the Internet and the rapid expansion of information technology, the signal age is making a subtle transition to the big data era. The network has turned into an integral part of people’s production and life. While enjoying the convenience brought by the information network, people also continuously feedback and input information to the network. Some information involves individual privacy, and network information security has become one of the hot topics of research. At present, the social network information security problem is becoming more and more obvious, the conventional information security software has been unable to deal with the endless information security problem, the network society urgently needs a new information technology to protect the increasingly huge information assets, and the big data technology has stronger insight, more scientific decision-making power and more accurate process optimization ability compared with the conventional software. Must be able to play a positive effect.

Professor Victor is known as the “Big Data Prophet.” Big data also called huge amount of data, refers to the amount of data involved is so large that it cannot be captured, managed, processed and collated in a reasonable time through the human brain or even mainstream software tools to help enterprises to make more positive decisions. By analyzing big data, we can draw conclusions that cannot be obtained in the case of small data. The big data we usually talk about is more about getting valuable information in a short time by quickly analyzing a large amount of data.

### Big Data Analysis Process and Features

**FIGURE 1:**
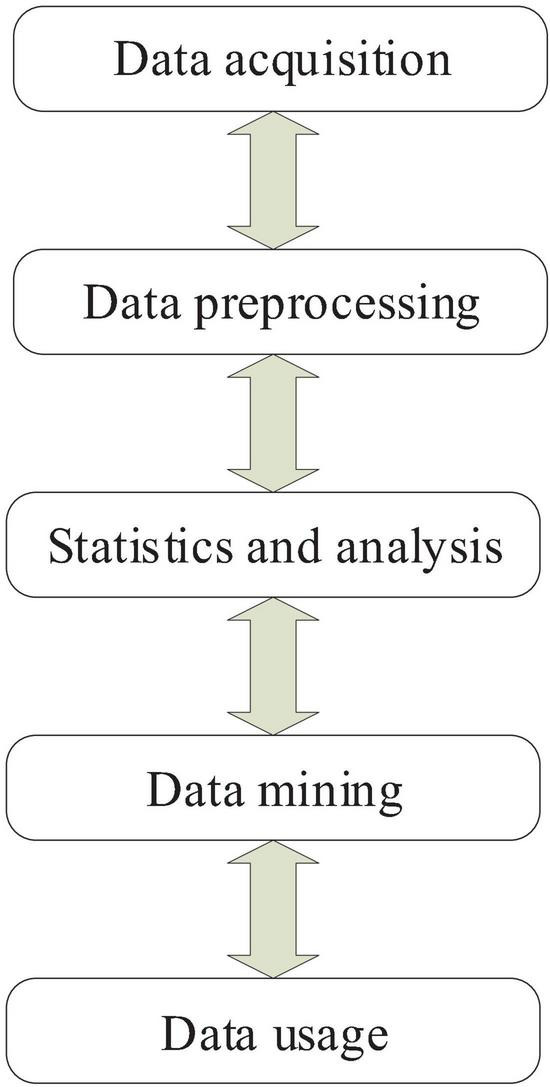
Big data processing flow.

In general, there are many methods for analyzing big data, and in theory it is still in the exploration stage, but no matter what kind of big data analysis method follows the basic process, the flow chart is shown in Figure 1.

The first process of big data analysis is acquisition. Big data sampling (Bivand and Krivoruchko, 2018; Cohen et al., 2018) means collect information collection platforms to collect users or other data. In the process of big data collection, the main problem is that the amount of data is huge, the amount of collection is large, and the data collection point is large. A large amount of data needs to be collected at the same time. Therefore, in the process of collecting big data, it is necessary to establish a larger database and how to further design the reasonable use and distribution of the database.

The second step is import and beneficiate. This mainly means that invalid information, redundant information and low-value information are excluded after the first information collection is completed, so it is necessary to execute the data before processing. Effective screening and brief analysis, and then import the resulting preliminary filtering information into another large database, this step is mainly to pre-process the big data.

The third step in big data processing is to perform statistics and analysis. This process is a process of further refinement of big data, analyzing and screening valid data, and performing statistical processing to obtain effective information.

The fourth step in big data is to deal with the mining (Rezaei-Hachesu et al., 2017; Fan et al., 2018; Svefors et al., 2019) process. Unlike the above process, there is no clear path or statistical analysis method for big data information mining. It is mainly used for databases that collect large amounts of data and use various algorithms for calculations, so it is complex data. Try to get predictions or get other valid conclusions. The statistical analysis and mining process of big data is considered to be a key process for transforming data from data into value space and value sources in the process of big data information processing.

The final step is using information obtained from big data. In particular, it can be used for business decision behavior predictions, while sales companies can provide accuracy. Marketing, achieving service conversion, etc. The application prospect of big data is very broad, and it has a good application prospect in transportation, sales management, economic research and forecasting.

At present, there is no authoritative unified standard. At present, the “4V” function of big data has been widely recognized.

First, the data size is huge. In 2012, the world produced about 2.7 billion GB of data per day, the amount of data per day equals the sum of all stored data in the world before 2000. Baidu must process more than 70,000 GB of search data per minute, and Alipay generates an average of 73,000 transactions per minute. Traffic flow monitoring systems and video capture systems can generate large amounts of video data at any time. Temperature sensors in greenhouses and various detectors in the factory are also big data manufacturers. It can be said that the amount of data we generate per minute is unimaginable. Now, the scale of data that big data needs to process continues to grow, reaching orders of magnitude unimaginable in small data.

Second, there is a wide variety of data (Variety). In big data, in addition to the ever-increasing data size, the types of data that people need to deal with are beginning to emerge. The various data types are very numerous and very strange, and only a few can be handled using traditional techniques. Some are unstructured data that traditional technologies cannot handle, and this trend will be long-term, with unstructured data accounting for 90% of all data over the next decade. For example, Tudou’s video library, photos on social networking sites, records, etc., even include RFID status, mobile operator call history, video surveillance video, Weibo and status posted on WeChat. The size, format, and type of data from various sources may vary. Existing data processing techniques are useless and can cause significant difficulties when performing large amounts of processing.

Third, value is difficult to mine. The first two features show that the amount of data and data types in big data are amazing. Faced with a large amount of data, in order to mine hidden “treasures,” the analysis and processing of powerful cloud computing systems is only one aspect, not even the main one. How to analyze big data from the perspective of innovation according to needs, what to use big data ideas to examine big data to explore unimaginable economic and social values. In other words, only the combination of technology and innovation can unlock the value of big data. Otherwise, no amount of data will be useful.

Fourth, the processing speed is high (Velocity). This is the most significant feature of the big data era, unlike the era of small data and the era of probability and statistics. In traditional economic censuses, censuses and other areas, data can be tolerated for days or even a year, as the data obtained at this time still makes sense. Moreover, due to technical limitations, the collected data has been lagging behind, and the structure of statistical analysis is lagging behind, but it must be accepted. Data generation and collection is very fast, and the amount of data is growing all the time. With advanced technology, people can collect data in real time. But in most cases, if you don’t process the data in time, the advanced collection and sorting methods will be meaningless and you won’t need big data. For example, IBM proposed the concept of “big data-level stream computing,” which is designed for real-time analysis of data and results to increase practical value. Therefore, timely and fast processing of data and results is the most important feature of big data.

This is the most significant feature of the big data, unlike the era of small data and the era of probability and statistics. Due to technical limitations, the collected data is backward, and the structure of statistical analysis is also backward, but it must be accepted. Data generation and collection is very fast, and the amount of data has been growing. With advanced technology, people can collect data in real time. But in most cases, if you don’t process the data in time, the advanced collection and sorting methods will be meaningless. For example, IBM proposed the concept of “big data-level stream computing,” which aims to analyze data and results in real time to increase practical value. Therefore, timely and fast processing of data and results is the most significant feature of big data.

### E-Commerce Concept

**FIGURE 2:**
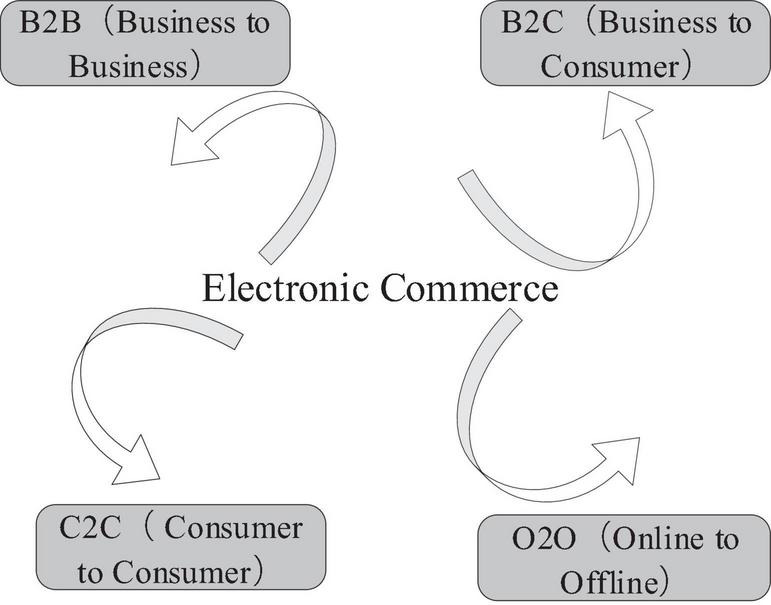
Main e-commerce model.

E-commerce generally refers to Internet technology, based on browser/server applications, through the Internet platform, buyers and sellers through various trade activities to achieve consumer online shopping, online payment and new business activities of various business activities and other models. The expansion history of e-commerce has a close relationship with the progress of computer network technology. E-commerce includes many models, such as B2B (Ning et al., 2018) (Business to Business), B2C (Business to Consumer), C2C (Consumer to Consumer), and O2O (Online to Offline). The main centralized e-commerce model is shown in Figure 2.

This article focuses on C2C (Sukrat and Papasratorn, 2018) e-commerce. C2C e-commerce refers to a network service provider that uses computer and network technology to provide e-commerce platforms and transaction processes to users in a paid or non-paid manner. Allow both parties to conduct online transactions on their platform. The two sides of the transaction are mainly individual users, and the trading method is based on bidding and bargaining. Like B2B and B2C, C2C is also a basic e-commerce transaction model. In real life, it is similar to the “small commodity wholesale market.” There are many self-employed people in a website, and the website’s role in e-commerce is equivalent to the “market manager” in actual market transactions. At the same time, in order to promote smooth transactions between buyers and sellers, C2C e-commerce provides a series of support services for both parties. For example, in cooperation with market information collection, credit evaluation systems and various payment methods have been established. Due to the rapid expansion of e-commerce, industrial agglomeration has become more impressive. The most prominent performance of industrial agglomeration is the industrial concentration of “Internet + traditional industries” such as “Taobao Village.” C2C is the mainstream of this e-commerce business model. C2C e-commerce “Taobao Village” is a product based on urban and rural expansion in China. It has Chinese characteristics and is a “Chinese product.” This is both a theoretical issue and a very real social phenomenon. The Chinese government has put forward the “Internet+” proposal. With the expansion of China’s strategic emerging industries, “Internet + traditional industries” will become a shortcut for China’s backward regions to seek expansion, which can shorten the time required for expansion, making C2C e-commerce a “hometown of Taobao.” Therefore, in order for the industry to complete transactions, an e-commerce platform and online and offline resources and services are needed. It can be said that the C2C model is an e-commerce model that is very suitable for industrial agglomeration. The biggest advantage of the C2C e-commerce model is that it can produce and deliver enterprise products or services on demand, so that enterprises can quickly develop into large enterprises, and the C2C e-commerce model provides consumers with cheap and affordable purchases. Product and service platforms enable businesses and consumers to achieve a win-win situation.

In traditional market transactions, the delivery of goods from producers to stores requires warehouse storage, vehicle transportation, etc., which increases inventory costs and transportation costs, resulting in increased transaction costs. Unlike real-world trading, since e-commerce joins the virtual network, both buyers and sellers trade through the e-commerce platform, so there is no need for face-to-face communication. This form saves the seller’s transaction costs, including physical store and merchandise inventory and transportation costs. At the same time, buyers can also shop without going out, and can quickly compare products of different merchants through the network, which allows buyers to get more information, more efficient and lower cost. C2C e-commerce uses Internet communication channels based on open standards. Compared with traditional communication methods (such as mail, fax, newspaper, radio, and television), communication costs are greatly reduced.

## Industry Agglomeration Virtual E-Commerce

### Industrial Cluster Concept

Industrial clusters attract the attention of many scholars by attracting resources, economies of scale, knowledge learning and innovation, saving transaction costs, and improving cooperation efficiency. Many mathematicians have studied the composition, characteristic mechanism, and identification criteria of industrial clusters through theoretical derivation, model construction, structural equations, and case studies, and elaborated and summarized the concept of industrial clusters. The definition of industrial agglomeration is that in a relatively limited space of a certain area, geographically adjacent or different geographical entities closely related to relevant institutions and government agencies spontaneously gather together, called industrial clusters. The division of labor between entities and continuous cooperation and innovation have formed a complex cluster network, providing environmental and technical support. The difference is that industrial clusters can adapt to economic expansion, and further transformation and upgrading will form a new industrial cluster model. At the same time, mutual trust, mutual decision-making, and close cooperation have created the greatest value and benefits for the industry. Finally, for the measurement and acquisition of industrial clusters, combined with the practical significance of empirical research, the measurement of industrial clusters is unified by the concentration of specific industries, that is, specific industries. A measure of the spontaneous aggregation of related entities or institutions in a particular industry in the region. If the total quantity or total capacity reaches the previous unified level, it indicates that there is an industrial cluster in the area.

### The Relationship Between E-Commerce and Industrial Clusters

With the rise and prosperity of e-commerce, the new business organization system breaks the regional and spatial barriers, promotes the use of e-commerce and partners, establishes synergy and sharing mechanisms, and continuously meets the needs of users. Proactively improve user experience and satisfaction. In addition, e-commerce platforms and logistics platforms are increasingly used in new business models. Although these platforms are very different, the role of the company cannot be underestimated. The platform typically includes several key functional modules such as trading markets, logistics platforms, enterprise services, cluster information, and corporate communities. Cluster companies can conduct informal technology and information exchange on the platform. Through the construction of an e-commerce platform, industrial cluster enterprises can share market conditions, the latest industry technologies, and related industry information in real-time and quickly, creating greater economic benefits for enterprises. This close partnership helps industry clusters increase trust and mutual benefit. In short, e-commerce applications can help industrial clusters effectively integrate regional resources, meet market demands promptly, expand clusters, and increase the level of collaboration and competitiveness of enterprises within the cluster. Currently, the introduction of e-commerce applications has further promoted the expansion of supply chain coordination. As an effective spatial organization model, industrial clusters play an increasingly important role in improving the overall economic level of the region and optimizing the allocation of industrial resources. The rapid expansion of industrial clusters provides natural conditions for the expansion of enterprises, between enterprises and between supply chain members. Similar companies continue to gather, and upstream and downstream companies in the supply chain are also gathered to promote the use of e-commerce. A deeper impact on the synergy of the supply chain. Therefore, for the sake of strengthening the application of e-commerce. Based on continuous research by many scholars, it is further proved that the rapid expansion of industrial clusters promotes the coordinated management of supply chains.

**FIGURE 3:**
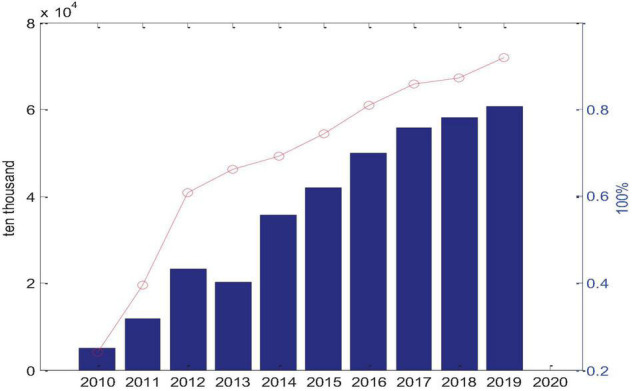
Number and proportion of mobile phone users.

Since 1980, the economy and the world have continued to develop. The Internet and information technology are constantly innovating. In addition to constantly affecting people’s daily lives in various aspects, it also leads the transformation of modern new production organizations. Figure 3 shows the statistics of Chinese netizens in the past decade. As can be seen from the above data, since the popularity of smartphones in 2013, mobile network users have occupied almost the entire network in the past 7 years. In the future expansion, mobile network users will develop more rapidly, making the popularity of mobile Internet and smart phones break the expansion of PC networks. At anytime, anywhere, and on the Internet, the online concept of the PC era has been broken, and immediacy has become a unique personality in the age of network information. A large amount of information, rapid response and scale effect are the main features of the e-commerce. The rapid spread of mobile phone business applications shows that the use of mobile phone networks by netizens has changed from basic communication entertainment to life entertainment. Since 2013, thanks to the expansion of domestic smart phone technology, the Internet access method based on mobile Internet has opened a new period of e-commerce and access to the Internet anytime and anywhere, so that more buyers and sellers can conduct transactions through the network, and Each transaction is based on online trading of various trading tools, and the trading platform and trading model have been rapidly developed. E-commerce has become a grassland, which has an impact on the production value chain, profit model and marketing methods of traditional industries. It can be seen that the growth of the network has boosted the expansion of e-commerce. The growth of e-commerce has promoted industrial agglomeration, and industrial agglomeration has formed economic globalization.

## Industrial Agglomeration Virtual E-Commerce Analysis

### China’s E-Commerce Transaction Scale

In the e-commerce environment, China’s e-commerce has undergone earth-shaking changes, especially in the past 30 years, the rapid growth of signal technology and technological innovation have made all aspects related to e-commerce stand out. The cost of online transactions has been greatly reduced, network communication is extremely convenient, and e-commerce is everywhere. On the basis of China’s national conditions, the application and expansion of e-commerce in China is different from that of other countries, but its expansion is in full swing. The expansion of China’s e-commerce is a signification part of accelerating the informationization of the national economy. At the same time, the application of e-commerce has also changed the production organization of enterprises to a large extent. Enterprises and users can interact directly with e-commerce related R&D, technology expansion, production, procurement, marketing and product operations. Other services and links can fully introduce user engagement and control market demand trends in real time. Table 1 shows the scope of China’s e-commerce transactions collected from the China E-Commerce Research Center.

**TABLE 1:** Scope of China’s e-commerce transactions.

	Transaction amount (100 million)	Growth (100 million)	Gain
2013	3.5	0.5	0.2
2014	4.35	0.85	0.242857143
2015	5.85	1.5	0.344827586
2016	7.63	1.78	0.304273504
2017	10.5	2.87	0.376146789
2018	13.35	2.85	0.271428571

**FIGURE 4:**
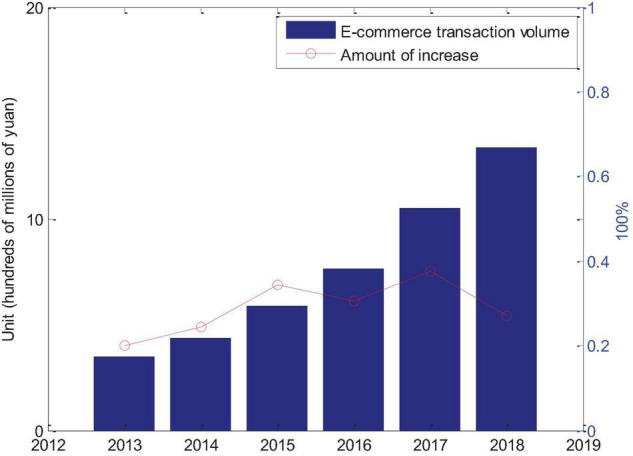
Trends in the scale of China’s e-commerce transactions.

Figure 4 shows the scope of China’s e-commerce market transactions from 2013 to 2017. It can be seen that as of 2018, China’s e-commerce still maintains a rapid growth trend. With the continuous encouragement and support of the government, all relevant systems are in a stage of continuous improvement. Under the impetus of e-commerce, enterprises and users, constantly proposing new consumer demand will help the rapid expansion of the upstream and downstream industry chains of traditional enterprises and provide new impetus for China’s economic expansion.

**FIGURE 5:**
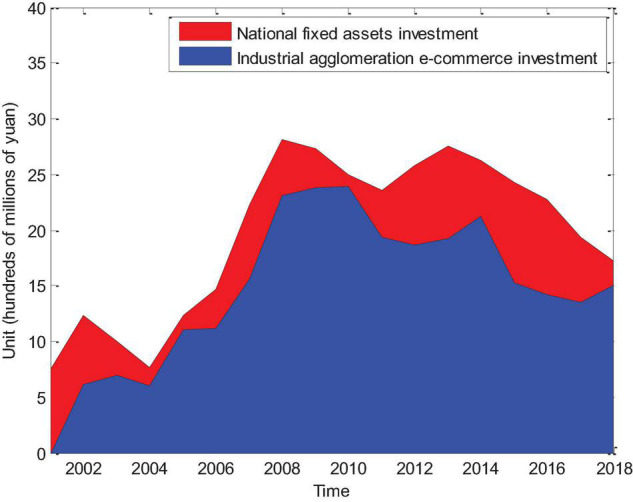
Changes in national fixed asset investment and industrial agglomeration e-commerce investment from 2001 to 2018.

It can be seen from Figure 5 that from 2001 to 2008, industrial agglomeration e-commerce investment and fixed asset investment are all levels of sustained growth, which proves that e-commerce expansion is relatively rapid during this period. In the future, industrial agglomeration investment profits can be Add a lot. From 2008 to 2015, the level of China’s economy was in a period of slow growth, and the investment level during this period was almost stable. After 2015, due to the saturation of the economy, the investment level remained at a certain level and the economic expansion region was stable.

### The Impact of the Level of Big Data Expansion on E-Commerce in Industrial Agglomeration

With the increasing popularity and expansion of the Internet, e-commerce has become an important aspect of Internet applications. In addition to the old e-commerce companies, traditional stores also opened their own online shopping malls. Consumers are also increasingly enjoying this convenient and fast way to shop. According to CNNIC’s statistical report, as of last year, the number of Internet users in China has reached 1.008 billion, and the proportion of online shopping among netizens has increased to 55.7%. In addition to online shopping, many service industries or national administrative departments have also increased the construction of online platforms, such as online car rental, travel route booking, room service, online transaction management fees, etc., further expanding the application field. E-commerce has created more business growth points. The expansion of business types and the explosive growth of business volume have brought a lot of data information. The old e-commerce companies Amazon, Alibaba and so on are all beneficiaries of big data. It can be said that without the support of big data technology, there is no e-commerce enterprise today.

The expansion of big data makes practitioners more competitive in e-commerce. From the perspective of the number of competitors, China’s e-commerce industry is currently in a highly concentrated stage. Although a large number of e-commerce companies have emerged, in the field of online retail, Taobao, Tmall, Jingdong, No. 1 store, Amazon and many others occupy most of the market. The emergence of big data has further increased barriers to entry, so the number of competitors in the online retail industry will change less. From the perspective of foreign competitors, it will undoubtedly increase the intensity of market competition. For example, the way Amazon enters the Chinese market is to acquire Joyo. From the perspective of switching costs, the e-commerce industry has typical low-cost conversion characteristics for consumers. On-site e-commerce companies often use large subsidies, promotions and free shipping to retain old users and win new users, which makes the market competitive. The pressure is constantly increasing. Pursuit of economies of scale. Most industries have significant economies of scale. E-commerce operators are pursuing economies of scale and blindly expanding, resulting in overcapacity, which ultimately led to fierce competition in the industry.

**FIGURE 6:**
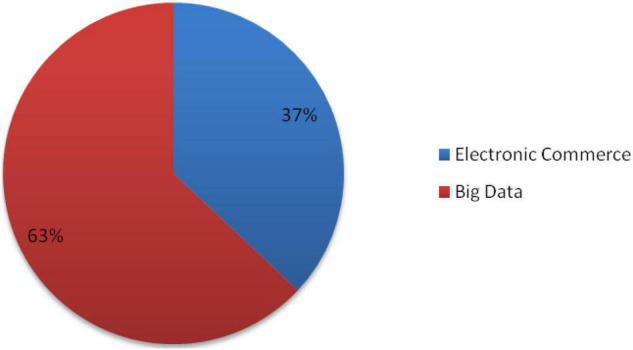
Impact of big data and e-commerce on industrial agglomeration expansion.

Figure 6 shows the expansion index for big data and e-commerce. As can be seen from the above data, in the future expansion process, big data is indispensable as a tool to support e-commerce and industrial agglomeration, and e-commerce is expanding very rapidly. The expansion of industrial agglomeration plays a very significant role. In the future, the expansion of e-commerce in all walks of life cannot be ignored. The future world is the electronic world and the data world. As an effective management mode of enterprise manufacturing and industrial organization, industrial cluster and supply chain management have become the inevitable requirements and strategic measures for enterprises to survive and develop in various fields. The coupled industrial cluster supply chain provides a new expansion trend for resource coordination and industrial upgrading, enabling cluster enterprises to improve traditional production methods, respond quickly to user needs, and consciously work closely together to grasp rapid changes more quickly and accurately. In order to deal with this problem, it is necessary to improve the operational efficiency of the enterprise through the information charge platform and modern management tools. Through the information platform, this work-use management becomes more complex, professional and standardized, thus freeing up enough energy to respond to industry changes.

In industrial agglomeration, the pioneering role of core enterprises should not be overlooked. If the pioneering enterprises can be cultivated effectively, through it to other enterprises and supporting enterprises to enter the industry to play a direct demonstration and produce cohesion, so that the formation of industrial agglomeration has a driving effect. At present, some of the core enterprises in the industry have already established a relatively complete e-commerce system. If we can combine the needs of SMEs in the industry, open up some of the functions of the system to a certain extent, and realize the sharing of information and knowledge with enterprises in the industry, this is very beneficial to enhancing the enthusiasm of SMEs to participate in industrial division of labor and cooperation, and at the same time lowering the this is very beneficial to increase the enthusiasm of SMEs to participate in industrial division of labor and cooperation, and at the same time reduces the threshold for SMEs to participate in e-commerce. A well-developed social network based on marketization or externalization is the basis for the formation and development of industrial clusters. To this end, the construction of information service organizations and networks within industrial clusters should be supported and encouraged to provide a variety of information services to enterprises, reducing the wasted costs and incomplete information caused by enterprises collecting information alone. At the same time, the construction of public institutions and means of communication that facilitate interaction between producers and the market should be strengthened, cooperation between enterprises and universities or research institutes should be encouraged, and the establishment of local public institutions that provide technical training, technical support and market information to producers should be supported. In addition, the construction of information advisory services should be accelerated and a multi-level public information platform should be established. In this regard, government departments or professional information service providers can intervene to provide a full range of information service approaches and dovetail with government public data platforms to achieve low-cost information services and knowledge provision within the industry.

### Analysis of Advantages and Disadvantages of Different Business Models in Industrial Agglomeration

**FIGURE 7:**
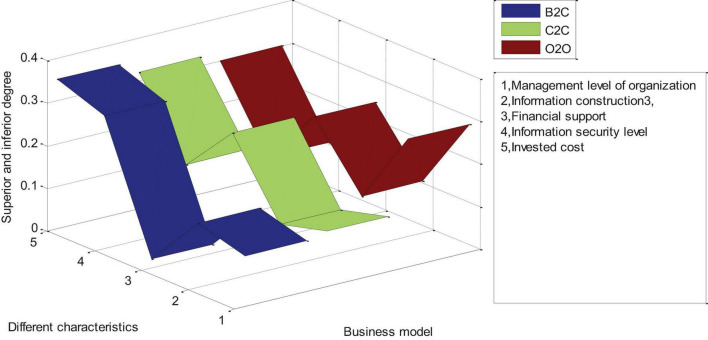
Advantages and disadvantages of different e-commerce in industrial agglomeration.

As shown in Figure 7, for the industrial agglomeration of the B2C e-commerce model, all goods and services of the enterprise are carried out through the network, including online shopping, online payment, logistics and after-sales. They are all done over the Internet and won’t be traded face to face. This model puts forward higher requirements for industrial agglomeration enterprises. Compared with the C2C and O2O models, the selection of the B2C e-commerce model requires that the industrial agglomeration area has a good organizational management level and complete information construction, because all activities are carried out online. Among the three e-commerce models, the B2C model has the highest information security requirements and requires more financial support and sufficient strength to ensure smooth transactions. For the C2C model, the needs of enterprises are much lower than those of B2C. For industries with insufficient funds, low level of enterprise informatization and low management level, C2C e-commerce model can be selected. The industrial cluster area builds an e-commerce trading platform through website construction. Consumers can find the trading objects and negotiate the transaction through the platform. Industrial agglomeration enterprises only need to optimize platform management, maintain transaction order, formulate transaction specifications, and improve trust mechanisms. Therefore, the C2C e-commerce model has lower requirements for the company’s capital, information and management level than the B2C model. For the O2O model, the network becomes the platform for offline transactions. For industrial clusters, the function of the C2C e-commerce model is to undertake the browsing work of consumers, let consumers understand the information through the platform, and then conduct transactions online. Therefore, it is necessary to reduce the investment cost of the C2C e-commerce model, and its management level and informatization level are lower than the B2C and O2O e-commerce models. Most industrial clusters can conduct business activities through the C2C platform.

## Conclusion

As the rising of Internet industry and other technologies, on the basis of the rapid expansion of e-commerce, the coordination problem of e-commerce has gradually emerged, affecting the organizational environment. At present, the research related to e-commerce and supply chain collaboration is getting more and more attention. As a new impetus for economic expansion, e-commerce has brought new impetus to the supply chain. In the process of supply chain coordination, e-commerce means making the required information more convenient and accurate, thus further enhancing the trust of the supply chain enterprises and the internal and external trust, and bringing economic benefits, the company has further expanded. In this paper, through the different applications of virtual e-commerce in industrial agglomeration, different e-commerce types highlight different characteristics in big data. Therefore, this paper analyses industrial agglomeration and electronics through literature comparison and data survey. The relationship between business, and through the investigation, we can see that the industrial agglomeration investment has been continuously expanded with the expansion of e-commerce and big data, which also proves that the future expansion of e-commerce is promising. Finally, the application of three different e-commerce models in industrial agglomeration is compared. The results show that different e-commerce models are determined by their own different, so we must choose the correct e-commerce model to adapt to the expansion of society through the actual situation.

Industrial agglomeration is an important way to enhance regional economic development, while e-commerce promotes the integration of enterprises into the world market. The author intends to analyse the problems of enterprise e-commerce in this context from the perspective of industrial agglomeration, and propose how to better realize the interaction between e-commerce and industrial agglomeration, so as to achieve the improvement of the competitiveness of enterprises in the industry.

## Data Availability Statement

The original contributions presented in the study are included in the article/supplementary material, further inquiries can be directed to the corresponding author.

## Author Contributions

HY was responsible for designing the framework of the entire manuscript from topic selection to solution to experimental verification.

## Conflict of Interest

The author declares that the research was conducted in the absence of any commercial or financial relationships that could be construed as a potential conflict of interest.

## Publisher’s Note

All claims expressed in this article are solely those of the authors and do not necessarily represent those of their affiliated organizations, or those of the publisher, the editors and the reviewers. Any product that may be evaluated in this article, or claim that may be made by its manufacturer, is not guaranteed or endorsed by the publisher.

## References

[B1] Anam F. Asad A. Wan M. Ahmad N. Z. (2017). Analyzing the academic research trends by using university digital resources: a bibliometric study of electronic commerce in China. *Univ. J. Educ. Res.* 5 1606–1613. 10.13189/ujer.2017.050918

[B2] Bivand R. Krivoruchko K. (2018). Big data sampling and spatial analysis: “which of the two ladles, of fig-wood or gold, is appropriate to the soup and the pot? *Stat. Probab. Lett.* 136 87–91. 10.1016/j.spl.2018.02.012

[B3] Cao Y. Xia Y. Cheng J. Huade Z. Yuancai C. (2017). A novel visible-light-driven In-based MOF/graphene oxide composite photocatalyst with enhanced photocatalytic activity toward the degradation of amoxicillin. *Appl. Catal. B Environ.* 200 673–680. 10.1016/j.apcatb.2016.07.057

[B4] Cohen M. C. Lobel R. Perakis G. (2018). Dynamic pricing through data sampling. *Prod. Oper. Manag.* 27 1074–1088. 10.1111/poms.12854

[B5] Fan C. Xiao F. Yan C. (2018). Research and applications of data mining techniques for improving building operational performance. *Curr. Sustain. Renew. Energy Rep.* 5 181–188. 10.1007/s40518-018-0112-x

[B6] Gui Y.-M. Wu Z. Gong B.-G. (2019). Value-added service investment decision of B2C platform in competition. *Kongzhi Juece Control Decis.* 34 395–405.

[B7] Han Y. Shao X. F. Tsai S. B. Fan D. Liu W. (2021). E-government and foreign direct investment: evidence from Chinese cities. *J. Glob. Inf. Manag. (JGIM)* 29 1–17. 10.4018/jgim.20211101.oa42

[B8] Heiner E. Marc G. (2018). Looking forward, looking back: British journal of management 2000-2015: looking forward, looking back. *Br. J. Manag.* 29 3–9. 10.1111/1467-8551.12257

[B9] Irene N. (2018). Review: electronic commerce. *ITNOW* 42 31–31.

[B10] Liu X.-F. Zhan Z.-H. Deng J. D. Li Y. Gu T. Zhang J. (2018). An energy efficient ant colony system for virtual machine placement in cloud computing. *IEEE Trans. Evol. Comput.* 22 113–128. 10.1109/tevc.2016.2623803

[B11] Nádudvari Á Fabiańska M. J. Marynowski L. Kozielska B. Konieczyński J. Smołka-Danielowska D. (2018). Distribution of coal and coal combustion related organic pollutants in the environment of the upper silesian industrial region. *Sci. Total Environ.* 62 1462–1488. 10.1016/j.scitotenv.2018.02.092 30045566

[B12] Navarro-Méndez D. V. Carrera-Suárez L. F. Sánchez-Escuderos D. Cabedo-Fabres M. Baquero-Escudero M. Gallo M. (2017). Wideband double monopole for mobile, WLAN, and C2C services in vehicular applications. *IEEE Antennas Wirel. Propag. Lett.* 16 16–19. 10.1109/lawp.2016.2552398

[B13] Nik B. Mark W. Ranjit S. Michael T. (2017). The use of microsoft excel as an electronic database for handover and coordination of patients with trauma in a district general Hospital. *BMJ Innov.* 3:130. 10.1136/bmjinnov-2016-000182

[B14] Ning J. Babich V. Handley J. Keppo J. (2018). Risk-aversion and B2B contracting under asymmetric information: evidence from managed print services. *Soc. Sci. Electron. Publ.* 66 392–408. 10.1287/opre.2017.1673 19642375

[B15] Rezaei-Hachesu P. Oliyaee A. Safaie N. Ferdousi R. (2017). Comparison of coronary artery disease guidelines with extracted knowledge from data mining. *J. Cardiovasc. Thorac. Res.* 9 95–101. 10.15171/jcvtr.2017.16 28740629 PMC5516058

[B16] Setiawan A. B. Dunan A. Mudjianto B. (2022). “Policies and innovations of financial technology business models in the digital economy era on the E-business ecosystem in indonesia,” in *Handbook of Research on Green, Circular, and Digital Economies as Tools for Recovery and Sustainability*, eds de Pablos Patricia O. Xi Z. Mohammad Nabil A. (Pennsylvania. PA: IGI Global), 22–42. 10.4018/978-1-7998-9664-7.ch002

[B17] Sukrat S. Papasratorn B. (2018). An architectural framework for developing a recommendation system to enhance vendors’ capability in C2C social commerce. *Soc. Netw. Anal. Min.* 8:22.

[B18] Suska M. (2022). “E-commerce: the pillar of the digital economy,” in *The European Union Digital Single Market*, eds Dabrowski L. D. Suska M. (Abingdon: Routledge), 63–91.

[B19] Svefors P. Sysoev O. Ekstrom E. C. Persson L. A. Arifeen S. E. Naved R. T. (2019). Relative importance of prenatal and postnatal determinants of stunting: data mining approaches to the MINIMat cohort, Bangladesh. *BMJ Open* 9:e025154. 10.1136/bmjopen-2018-025154 31383692 PMC6687011

[B20] Wang K. Xu C. Yan Z. Guo S. Zomaya A. (2017). Robust big data analytics for electricity price forecasting in the smart grid. *IEEE Trans. Big Data* 5 34–45. 10.1109/tbdata.2017.2723563

[B21] Wang X.-S. Yu C.-Y. (2017). Impact of spatial agglomeration on industrial pollution emissions intensity in China. *Zhongguo Huan. Kexue China Environ. Sci.* 37 1562–1570.

[B22] Xuan S. U. N. (2017). Multi-indicator evaluation and analysis of coordinated industrial expansion of urban agglomerations. *Urban Environ. Stud.* 05:1750006. 10.1142/s2345748117500063

[B23] Yang L. Zheng Y. Y. Wu C. H. Dong S. Z. Shao X. F. Liu W. (2022). Deciding online and offline sales strategies when service industry customers express fairness concerns. *Enterp. Inf. Syst.* 16, 427–444.

[B24] Yu P. Gong R. Sampat M. (2022). “Blockchain technology in china’s digital economy: balancing regulation and innovation,” in *Regulatory Aspects of Artificial Intelligence on Blockchain*, ed. Pardis Moslemzadeh T. (Pennsylvania. PA: IGI Global), 132–157. 10.4018/978-1-7998-7927-5.ch007

[B25] Zhou Y.-Z. Li P.-X. Wang S.-G. Xiao F. Li J. Z. Gao L. (2017). Research progress on big data and intelligent modelling of mineral deposits. *Bull. Miner. Petrol. Geochem.* 36 327–331.

